# Surveying alcohol and other drug use through telephone sampling: a comparison of landline and mobile phone samples

**DOI:** 10.1186/1471-2288-13-41

**Published:** 2013-03-16

**Authors:** Michael Livingston, Paul Dietze, Jason Ferris, Darren Pennay, Linda Hayes, Simon Lenton

**Affiliations:** 1 Drug Policy Modelling Program, National Drug and Alcohol Research Centre, University of New South Wales, Sydney, Australia; 2 Centre for Alcohol Policy Research, Turning Point Alcohol and Drug Centre, Melbourne, Australia; 3 Centre for Population Health, Burnet Institute, Melbourne, Australia; 4 ARC Centre for Excellence in Policing, Brisbane, Australia; 5 Institute for Social Science Research, University of Queensland, Queensland, Australia; 6 Social Research Centre, Melbourne, Australia; 7 Centre for Behavioural Research in Cancer, Cancer Council Victoria, Victoria, Australia; 8 National Drug Research Institute, Curtin University, Perth, Australia

## Abstract

**Background:**

Telephone surveys based on samples of landline telephone numbers are widely used to measure the prevalence of health risk behaviours such as smoking, drug use and alcohol consumption. An increasing number of households are relying solely on mobile telephones, creating a potential bias for population estimates derived from landline-based sampling frames which do not incorporate mobile phone numbers. Studies in the US have identified significant differences between landline and mobile telephone users in smoking and alcohol consumption, but there has been little work in other settings or focussed on illicit drugs.

**Methods:**

This study examined Australian prevalence estimates of cannabis use, tobacco smoking and risky alcohol consumption based on samples selected using a dual-frame (mobile and landline) approach. Respondents from the landline sample were compared both to the overall mobile sample (including respondents who had access to a landline) and specifically to respondents who lived in mobile-only households. Bivariate comparisons were complemented with multivariate logistic regression models, controlling for the effects of basic demographic variables.

**Results:**

The landline sample reported much lower prevalence of tobacco use, cannabis use and alcohol consumption than the mobile samples. Once demographic variables were adjusted for, there were no significant differences between the landline and mobile respondents on any of the alcohol measures examined. In contrast, the mobile samples had significantly higher rates of cannabis and tobacco use, even after adjustment. Weighted estimates from the dual-frame sample were generally higher than the landline sample across all substances, but only significantly higher for tobacco use.

**Conclusions:**

Landline telephone surveys in Australia are likely to substantially underestimate the prevalence of tobacco smoking by excluding potential respondents who live in mobile-only households. In contrast, estimates of alcohol consumption and cannabis use from landline surveys are likely to be broadly accurate, once basic demographic weighting is undertaken.

## Background

Licit and illicit drug use is linked to a wide range of negative health and social outcomes [1-3] and has become a key concern for public health research. Survey research is a key component of the work in this area, with population surveys used to measure prevalence of use and risky use, to assess predictors of use and to monitor trends over time e.g. [4-7].

A key limitation of survey methods relates to the potential bias due to non-response. This occurs when respondents to a particular survey differ systematically from non-responders [8]. A number of studies have demonstrated that people who choose not to respond to surveys or who take more effort to recruit report higher rates of tobacco, alcohol and illicit drug use [9-12]. Increasingly, there are concerns that further bias is being introduced into survey estimates of these behaviours by the exclusion of otherwise eligible respondents who are not contactable because they do not have access to a landline telephone.

A substantial amount of health survey research makes use of samples drawn from landline telephone numbers using random digit dialling (RDD) [5,7]. However, there are increasing concerns about the representativeness of samples selected using landline RDD sampling frames due to the rapidly increasing number of households without landlines [13]. In 2011, estimates reported by the Australian Communications and Media Authority [14] suggest that around one-fifth of adults live in mobile phone only households and would thus be excluded for any surveys based on landlines alone. Internationally, this issue is a growing concern – around a third of households in the USA have no landline [15], while mobile-phone only households outnumber those with a landline in at least nine countries in the EU [16].

These changes in the representativeness of landline-based samples will result in substantial non-response bias in survey estimates if there are significant behavioural differences between those that are accessible via landline telephone and that are not.

In the USA, the recent decline in the prevalence of binge drinking, heavy alcohol consumption and smoking among young adults found in the Behavioural Risk Factor Surveillance System (a landline-based RDD telephone survey) has been attributed to the increasing numbers of young people living in mobile-only households and thus excluded from the sampling frame [17]. Only a handful of studies have directly compared estimates of health behaviours derived from mobile-phone and landline samples. Hu et al. [18] estimated that landline samples in the USA underestimate the prevalence of heavy episodic drinking by 14.8% and of smoking by 10.3%, even after adjustment for age, sex and education levels. Similarly, Blumberg and Luke found significantly higher rates of heavy drinking and smoking among a sample of young adults reached on mobile phones, compared with a similar landline based sample [19].

This issue has received less attention in Australia. A face-to-face study of South Australian households between 1999 and 2008 identified a steady increase in the number of mobile-only households, and that increases were particularly sharp amongst young, socio-economically disadvantaged people [20]. A small study of young women compared the prevalence estimates of a range of sexual health related outcomes from mobile- and landline-recruited samples, finding few differences [21]. In a more comprehensive study, Pennay [22] studied the differences between mobile-only and landline households in Australia. Even after weighting the data to account for the different underlying age, sex and region distributions of the two samples, the mobile-only sample had higher prevalence estimates of risky drinking, smoking, illegal drug use and problem gambling. Therefore, this study provided some evidence that undercoverage in landline-based surveys focussed on risky health behaviours is an important issue.

In this study we examine whether there are any differences in the estimates of alcohol, tobacco and illicit drug use obtained from landline versus mobile phone sampling strategies. Importantly, we consider these differences across a range of outcomes and in the context of key control variables typically used for sample weighting in survey research (age, sex, educational attainment). In light of previous research on this issue we expect that our mobile-phone sample would be more likely to report health risk behaviours. We have analysed our data in two ways – firstly comparing the respondents living in mobile-only households with the landline sample and then comparing the prevalence estimates obtained from the dual-frame survey^a^ with those from the landline component only to determine the size of any bias introduced by only sampling landline households. This strategy enables us to answer two key questions: 1) How different are mobile-phone only respondents from respondents sampled using traditional CATI methods?, and 2) How well does the use of a dual-frame sampling regime overcome issues of undercoverage in landline-based telephone surveys?

## Methods

### Survey methods

A commercial sample provider, Sampleworx, was contracted to provide two sampling frames of adult Australians (18 years and over), landline and mobile. Selection probability for the landline frame was set through size quotas for the capital city and non-capital city regions of each state/territory (the Australian Capital Territory was treated as one region). In Australia there are no geographic identifiers available for mobile phones, and so a simple national random sample frame was devised.

Calls to landlines and mobiles were made by trained interviewers from the Social Research Centre in Melbourne. Respondents to the landline calls were asked to provide access to the person in the household whose birthday was next due, while the mobile phone respondent themselves was assessed for eligibility. A range of techniques were used to boost response rate, including using voicemail if there was no answer, using bilingual interviewers to meet demand for interviewing in common languages other than English, and calls to immediate hang-ups and ‘soft-refusals’ at the discretion of the interviewer.

A total of 76,342 calls (28,070 landline and 48,272 mobile) resulted in a final sample of 2014 (1012 landline, 1002 mobile phone). This represents an overall response rate of 16%, according to AAPOR standard Response Rate 3 definition [23]. The response rate varied between sampling frames with a response rate of 22% for landlines and 13% for mobiles. More calls were needed to recruit the mobile sample because mobile calls (33%) went more frequently to answering services than landline calls (14%). Successful calls saw the administration of a structured questionnaire by interviewers that covered a range of health-related issues.

Ethics approval was obtained from the University of Queensland, Behavioural and Social Sciences Ethical Review Committee.

### Outcome measures

Data on alcohol consumption were collected using a series of four questions covering the 12 months prior to the survey, including simple two-question usual quantity/usual frequency items [24] and two additional questions asking the respondent how often they consumed five or more and eleven or more standard drinks in a single drinking session. The first two questions were used to derive an estimate of annual drinking volume (frequency of drinking multiplied by usual amount), while the second two were dichotomised to provide measures of whether or not the respondent had consumed 5+ (henceforth ‘risky drinking’) and/or 11+ (henceforth ‘very-risky drinking’) in the last 12 months.

Smoking status was assessed using a single standard item which asked how often respondents currently smoked cigarettes, pipes, cigars or any other tobacco products. This item was dichotomised into current smokers (anyone who reported any current smoking, including those who smoked less than weekly) and non-smokers [25].

Cannabis use was measured using two questions: firstly asking whether the respondent had ever used cannabis and secondly if they had used it in the last 12 months. Both of these items were analysed as simple dichotomous variables (‘lifetime cannabis use’ and ‘recent cannabis use’).

### Other measures

Sample type was defined in two ways to answer the two key questions detailed above. Firstly as a dichotomous variable, with respondents either coming from the mobile-phone sampling frame or the landline sampling frame and secondly with respondents from mobile-only households compared with the landline sampling frame (respondents from the mobile-phone sampling frame who reported having access to a landline were excluded from analyses using this variable).

A number of other control variables were selected for analyses: sex (male, female), age (18-24, 25-39, 40-49, 50-64, 65+), location (metropolitan, regional/rural), and education level (less than Year 10, Year 10, completed high school, trade qualification or diploma, university degree) as these are often used in sample weighting.

### Analysis

Unless specified, analyses were based on weighted data. Weights incorporated an adjustment accounting for the likelihood of respondents being sampled and post-stratification weighting based on the age, sex, region, telephone status (landline only, dual-user, mobile only) and educational attainment of the sample (matched to population distributions from the Australian Census of Population and Housing, [26]). Weighting was undertaken on the overall sample and separate weights were also produced for the landline and mobile samples separately.

Crude prevalence estimates of risky drinking, very risky drinking, smoking, lifetime and recent cannabis use as well as a measure of the total volume of alcohol consumed were calculated and compared across the unweighted samples. Differences were considered significant if the 95% confidence intervals of the estimates did not overlap. Further analyses were then undertaken to examine whether any differences between the samples could be explained by differences in their demographic structures. These involved logistic (dichotomous outcomes) and negative binomial (annual alcohol volume) regression models that were developed for each of the six outcome variables discussed above, controlling for age-group, sex, location and educational attainment. All analyses were conducted using Stata v12.1 [27]. Negative binomial regression was used for the annual alcohol volume outcome as it is count data, rather than a dichotomous measure. The interpretation of the incidence-rate ratio (IRR) produced by this regression is similar to that of the odds ratios presented for logistic regression – the IRR represents the relative increase (or decrease) in total drinking for the group under analysis in comparison with the reference group.

## Results

Unweighted descriptive statistics for each of the two sampling frames and the mobile-only subsample are presented in Table 1, along with population estimates for age, sex and region from the 2011 Census [26] and from the combined total dual-frame sample for comparison (note that highest level of education is not provided in the Australian Census, and is thus not included here). There are substantial differences. The mobile-phone sample had a higher proportion of males, a younger age distribution and more people from metropolitan areas. The landline sample was slightly less educated, with a significantly higher proportion of respondents who had not completed high school and a lower proportion with university qualifications. Compared to the population estimate, the mobile samples were comprised of a higher proportion of males and young people, while the landline sample were significantly older and more likely to be female. More than 70% of people in the mobile-only subsample were younger than forty, compared with 39% of the population and 23% of the landline sample. The combined dual-frame sample is the most representative of Census data, with age and metro distributions very similar to population distributions.

**Table 1:** **Unweighted descriptive statistics of mobile and landline samples and mobile**-**only subsample**

	**Mobile sample**	**Landline sample**	**Mobile**-**only subsample**	**Combined sample (dual**-**frame)**	**Population estimate (census data)**
N (unweighted)	1002	1012	295	2014	
*Gender*					
Male	52% (49%-55%)*	37% (34%-40%)	57% (51%-63%)*	56% (53%-58%)	49%
*Age group*					
18-24	20% (18%-23%)*	3% (2%-5%)	23% (18%-28%)*	12% (10%-13%)	12%
25-39	33% (30%-36%)*	15% (13%-18%)	48% (42%-54%)*	24% (22%-26%)	27%
40-49	16% (14%-18%)	18% (16%-21%)	9% (6%-12%)*	17% (15%-19%)	18%
50-64	24% (21%-26%)*	31% (28%-34%)	16% (11%-20%)*	28% (26%-30%)	24%
65+	7% (5%-9%)*	32% (29%-34%)	4% (2%-7%)*	19% (18%-21%)	18%
*Region*					
Metro	72% (69%-74%)*	64% (61%-67%)	70% (65%-75%)	68% (66%-70%)	68%
*Education level*					
< year 10	5% (4%-7%)*	11% (9%-13%)	6% (3%-8%)*	8% (7%-9%)	
Year 10	13% (11%-15%)*	19% (17%-22%)	14% (10%-18%)	16% (15%-18%)	
Completed high school	21% (19%-24%)*	17% (15%-19%)	18% (14%-23%)	19% (17%-21%)	
Trade qualification/diploma	24% (22%-27%)	24% (22%-27%)	31% (25%-36%)	24% (23%-26%)	
Degree or higher	35% (33%-38%)*	28% (26%-31%)	31% (26%-36%)	32% (30%-34%)	
*Outcome variables*					
Current drinker (last 12 months)	83% (80%-85%)	82% (80%-84%)	79% (75%-84%)		
Total alcohol volume estimate (standard drinks)	317 (286-349)	279 (248-311)	357 (298-416)		
Risky drinking (5+std drinks)	57% (54%-60%)*	41% (38%-44%)	60% (55% - 66%)*		
Very-risky drinking (11+ std drinks)	30% (27%-33%)*	16% (13%-18%)	36% (30%-41%)*		
Recent cannabis use	12% (10%-13%)*	3% (2%-5%)	17% (13%-22%)*		
Lifetime cannabis use	41% (38%-44%)*	29% (26%-31%)	49% (44%-55%)*		
Current smoker	23% (21%-26%)*	14% (12%-16%)	34% (28%-39%)*		

* Significantly different from the landline sample (based on survey-derived 95% confidence intervals).

The total mobile and landline samples were significantly different on all of the outcome variables except for drinking volume, with the total mobile sample reporting higher rates of risky and very-risky drinking, lifetime and recent cannabis use and current smoking. The mobile-only subsample was even more different to the landline sample, particularly on the cannabis and tobacco measures. It is worth noting that the mobile-only sample is small, and thus estimates for it have a substantial amount of uncertainty (as seen by the wide confidence intervals in Table 1).

To test whether these differences in the outcome variables simply reflected the markedly different demographic structures of the samples a series of regression models, controlling for key demographic variables were developed. Firstly, we examined the differences between the mobile-phone only subsample and the landline sample, to assess whether respondents who cannot be accessed using traditional landline approaches differ significantly from those who can. The bivariate relationships between telephone status (mobile-only vs landline) and each of the outcomes from these models are presented in Table 2.

**Table 2:** **Relationship between telephone status**, **drinking**, **cannabis use and smoking**

	**Adjusted odds ratio for mobile-only subsample (compared to landline sample)**	**95% Confidence interval**
Total drinking volume^+^	1.15	(0.88-1.51)
Risky drinking	0.90	(0.62-1.29)
Very risky drinking	1.11	(0.76-1.62)
Lifetime cannabis use	1.55	(1.09-2.20)*
Recent cannabis use	2.36	(1.30-4.30)*
Current smoking	2.43	(1.65-3.57)*

Controlling for age, sex, location and education status.

* statistically significant at p < 0.05.

^+ ^Note that the model for total volume was a negative binomial regression model, and the parameter presented here for it is an Incident Rate Ratio rather than an Odds Ratio.

There were no significant differences in drinking behaviour between the mobile-only subsample and the landline sample after adjustment for basic demographic variables. In contrast, rates of lifetime and recent cannabis use and current smoking were significantly higher in the mobile-only subsample.

To further explore the impact of interviewing methods on estimates of risky behaviour prevalence, prevalence estimates for each of the behaviours examined above were calculated separately for the landline, mobile and combined samples. This approach allows us to examine the impact of incorporating a mobile-phone sampling frame into studies aimed at estimating health risk behaviour rates, and provides an indication on prevalence estimates of relying on landline telephones to measure risk behaviours. The prevalence estimates for each of the outcome variables using each sample are presented in Table 3.

**Table 3:** **Weighted prevalence estimates drinking**, **cannabis use and smoking by sample type**

	**Mobile sample**		**Landline sample**		**Dual-frame sample**	
	**Prevalence**	**95% CI**	**Prevalence**	**95% CI**	**Prevalence**	**95% CI**
Risky drinking	55%	(52% - 59%)	51%	(47% - 55%)	52%	(49% - 54%)
Very risky drinking	28%	(25% - 32%)	24%	(20% - 28%)	26%	(24% - 28%)
Lifetime cannabis use	40%	(37% - 44%)	35%	(31% - 40%)	37%	(35% - 40%)
Recent cannabis use	10%	(8% - 12%)	8%	(4% - 11%)	9%	(7% - 10%)
Current smoking	24%	(21% - 28%)*	16%	(13% - 19%)	21%	(19% - 23%)*

* Significantly different from landline sample prevalence estimate.

The mobile and dual-frame samples produced higher prevalence estimates than the landline sample across all of the behaviours studied. However, the differences in estimates between the landline sample (as traditionally used in survey research) and the dual-frame sample utilised in this study were generally small and non-significant, with significant differences in prevalence only found for current smoking (16.1% in the landline sample, compared with 21.0% in the dual-frame sample).

## Discussion

The results of this study show that estimates of the prevalence of licit and illicit drug use vary between RDD samples recruited through mobile phones and landlines. In particular, rates of cannabis use and current tobacco smoking are significantly higher among respondents who cannot be accessed using traditional landline-based telephone surveys. This mobile-phone only segment of the population makes up around one-fifth of Australian households, and disproportionately includes young males, a traditionally hard to reach demographic in survey research [28]. Initial analyses found that much of the differences between the mobile and landline samples could be explained by demographic factors, although the mobile-only population had significantly higher rates of cannabis and tobacco use even after adjusting for age, sex, location and education status. In contrast the mobile-only sample showed no significant differences in drinking behaviour once these factors were adjusted for. The contrast between alcohol and cannabis and tobacco is puzzling. The differences do not appear to be related simply to the relative prevalence of the different behaviours, as these were all within similar ranges (except perhaps for recent cannabis use). Cannabis and to a lesser extent tobacco use are more stigmatised than alcohol use in Australian society, with the 2010 National Drug Strategy Household Survey estimating that 45% of Australians approved of regular alcohol use, compared with 15% for tobacco and 8% for cannabis [4], p157, but how such cultural effects relate to the differences in response across samples is unclear.

When examining overall prevalence estimates, the results presented here show that landline survey methods generally underestimate the prevalence of risky behaviours. However, the differences between the landline and dual-frame samples were relatively small (and non-significant) for measures relating to alcohol and cannabis suggesting that, with appropriate weighting, landline survey methods can provide relatively robust estimates of these behaviours. In contrast, the use of a dual-frame sample incorporating mobile-phones produced significantly higher prevalence estimates of smoking behaviour, meaning that landline-based appear to be particularly likely to underestimate smoking prevalence.

The results presented here demonstrate the utility of a dual-frame survey approach incorporating a mobile telephone sample for providing optimal estimates of tobacco use at the population level. It is also clear that our dual-frame sample provided a more representative sample demographically than either the mobile or landline samples on their own. However, this study doesn’t demonstrate marked improvements from using a dual-frame sample over a landline sample for other health-risky behaviours (risky drinking and cannabis use). It is also worth noting that there remain methodological issues with conducting population surveys via mobile phone, which is reflected in our low response rate (13%) for this study. Future studies should also incorporate larger mobile-only samples to ensure robust estimates on this key population group.

These findings differ from previous results (all from the USA), where significant differences in estimates of alcohol consumption between mobile and landline samples have been found e.g. [18,19] even when controlling for a selected demographics. This may in part reflect the higher proportion of landline-free households in the USA, suggesting the need for further research in future as distribution of telephone access in Australia changes over time. In contrast, the findings for tobacco are more consistent with the previous literature on health risk behaviours. We could locate no previous study examining illicit drug use. Based on the results found in this study, estimates of tobacco smoking using landline-based telephone survey methodologies will significantly underestimate prevalence rates by excluding respondents in mobile-only households who undertake these behaviours more frequently than people living in households with landline telephones.

## Conclusions

Overall, the findings of this study suggest that the undercoverage bias specifically resulting from using landline sampling frames to measure risky alcohol consumption is small and non-significant (undercoverage and non-response bias in surveys of drinking and drug use are broader problems that clearly lead to underestimates of these behaviours [11,12]). Similarly, while there were significant differences between the samples for cannabis use in logistic regression models, prevalence estimates from the landline sample were not significantly different to those based on the dual-frame data. However, the findings for tobacco, combined with recent Australian data showing steady increases in the proportion of households without a landline (14), mean that increasing attention will need to be paid to ensuring that surveys aiming to measure health behaviours adequately cover households without landlines. This can be done using the approach outlined in this study (a dual-frame telephone survey) or via face-to-face or other non-telephone based survey approaches [20].

### Endnote

^a^ i.e. the combined mobile and landline samples.

## Competing interests

DP is Managing Director and Head of Research Strategy at the Social Research Centre P/L, a social research company with specialist expertise in survey research. The key questions analysed in the study were designed by the researchers and the SRC played no role in the analysis of the data for this study, beyond the authorship contribution of DP.

## Authors’ contribution

All authors were involved in the conception and design of the study. DP had oversight of the overall data collection and survey methods. ML, PD and JF undertook the statistical analyses. ML and PD managed the initial drafting of the manuscript. All authors contributed to and have approved the final version.

## Pre-publication history

The pre-publication history for this paper can be accessed here:

http://www.biomedcentral.com/1471-2288/13/41/prepub
